# Laboratory and functional tests in leprosy diagnosis: a practical guide for clinical decision-making^؆^

**DOI:** 10.1016/j.abd.2026.501297

**Published:** 2026-03-20

**Authors:** Luis Alberto Ribeiro Fróes, Mirian Nacagami Sotto, José Antônio Garbino, João Avancini, Maria Ângela Bianconcini Trindade

**Affiliations:** a Department of Pathology, Faculdade de Medicina, Universidade de São Paulo, São Paulo, SP, Brazil; b Department of Dermatology, Faculdade de Medicina, Universidade de São Paulo, São Paulo, SP, Brazil; c Instituto de Medicina Tropical, Universidade de São Paulo, São Paulo, SP, Brazil; d Instituto Lauro de Souza Lima, Área de Neurofisiologia Clínica, Bauru, SP, Brazil

**Keywords:** Diagnosis, Diagnostic techniques and procedures, Leprosy, *Mycobacterium leprae*, Peripheral nervous system diseases, Ultrasonography

## Abstract

Early diagnosis of leprosy remains primarily clinical, based on recognition of skin lesions with altered sensation and peripheral nerve involvement. However, complementary tests play crucial roles in confirming uncertain cases, guiding operational classification, detecting subclinical neural involvement, and distinguishing late reactions from relapse. This review synthesizes the practical application of diagnostic methods available to dermatologists. Slit-skin smear microscopy demonstrates high specificity but limited sensitivity in paucibacillary forms, while histopathology reveals the characteristic immunopathological spectrum, with perineural acid-fast bacilli being pathognomonic for leprosy. Molecular detection by PCR enhances diagnosis in paucibacillary cases (34%‒80% sensitivity) but cannot distinguish viable from non-viable bacilli, limiting its utility in post-treatment assessment. Anti-PGL-1 serology aids contact surveillance, with seropositive individuals showing 3.5-fold increased risk of developing disease, though sensitivity remains below 30% in tuberculoid forms. For neural evaluation, Semmes-Weinstein monofilament testing provides a standardized tactile threshold assessment, while the histamine test maps autonomic dysfunction, particularly valuable in indeterminate forms. Electrodiagnostic studies reveal early subclinical changes and monitor reaction-related neural deterioration. Peripheral nerve ultrasonography demonstrates superior sensitivity over palpation for detecting thickening (97.4% vs. 30% concordance) and identifies inflammatory activity through Doppler assessment. When evaluating post-treatment complications, an integrated approach combining bacteriological index trajectory, histopathological patterns, PCR cycle threshold values, and serological trends enables reliable differentiation between therapeutic failure, late reactions, and relapse. No single laboratory test confirms early leprosy in isolation; clinical dermato-neurological expertise remains paramount, with complementary tests interpreted within the epidemiological context to optimize diagnostic accuracy and therapeutic decisions.

## Introduction

Globally, leprosy remains a public health challenge, with most new cases concentrated in India, Brazil, and Indonesia. Early and accurate diagnosis is essential to interrupt the chain of transmission and prevent permanent disability. Diagnosis is primarily clinical, based on recognizing skin lesions with altered sensation and peripheral nerve involvement. Early signs include areas of thermal hypoesthesia, paresthesias, reduced hair density, and diminished sweating, which often precede more evident manifestations.

In the initial and paucibacillary forms ‒ precisely when intervention is most effective ‒ careful history-taking and a systematic dermato-neurological examination are paramount. Semmes-Weinstein monofilament testing and the histamine test are the most practical point-of-care assessments to confirm sensory and autonomic dysfunction in these presentations.^1^ Laboratory investigations ‒ slit-skin smear microscopy, histopathology, molecular detection, serology, and imaging ‒ play a complementary role, with sensitivities that vary by clinical form. Importantly, these methods have significant limitations in early disease when they would be most needed.

Judicious selection and proper interpretation of these methods help confirm uncertain cases, guide operational classification, detect subclinical neural involvement, distinguish reactional episodes from relapse, and investigate drug resistance. This review synthesizes the main complementary diagnostic tests ‒ their indications, techniques, interpretation, and diagnostic pitfalls ‒ offering practical guidance to optimize laboratory investigation while maintaining the primacy of clinical examination.

## Slit-skin smear examination (bacilloscopy)

Slit-skin smear examination is a laboratory technique that detects *M. leprae* in intradermal scrapings and estimates the patient's bacillary load. The procedure involves superficial skin incisions with sampling from standardized sites ‒ right and left earlobes, right and left elbows ‒ and, when present, one active skin lesion. The Bacteriological Index (BI) follows Ridley's logarithmic scale, ranging from 0 (no bacilli in 100 fields) to 6+ (> 10³ bacilli per field). The mean of indices obtained from the different sites provides an estimate of the patient's overall bacillary burden.^2^

For staining, the modified Ziehl-Neelsen (cold) method is used because it preserves bacillary morphological characteristics. Morphological assessment complements quantification: uniformly stained bacilli suggest viability, whereas fragmented and granular forms indicate non-viable organisms. Globi ‒ aggregates of 30 to 100 bacilli embedded in lipid material ‒ correlate with multibacillary disease with high bacillary loads (Brazil, 2010). After treatment, fragmented or granular bacilli represent non-viable bacillary remnants undergoing slow clearance and do not indicate therapeutic failure. The BI typically declines by approximately 0.85 log units per year, with a steeper decrease in the first 24-months (approximately 1.15 log/year).^3^ This gradual decline renders the test unsuitable for early monitoring of treatment response.^4^

Although demonstrating high specificity and low cost, with relatively straightforward execution when performed by trained personnel, test sensitivity depends directly on bacillary load. Detection of Acid-Fast Bacilli (AFB) in slit-skin smears not only confirms the diagnosis but also automatically classifies the patient as multibacillary, regardless of the number of skin lesions.^2^ The examination is particularly valuable for establishing baseline BI at treatment completion in multibacillary patients, providing an essential reference for future comparisons when relapse is suspected.

### Histopathological examination

**Fig. 1:**
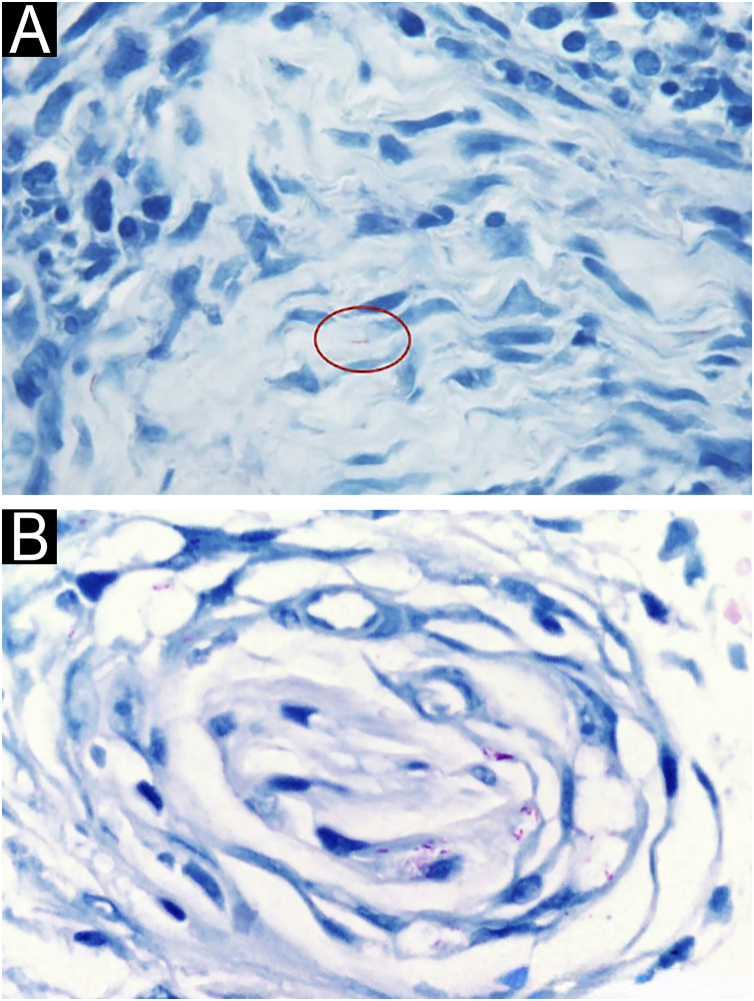
Acid-fast bacilli in cutaneous nerves. (A) Paucibacillary cutaneous nerve with an acid-fast bacillus within a Schwann cell (Fite-Faraco, ×100). (B) Multibacillary cutaneous nerve (transverse section) showing numerous acid-fast bacilli within Schwann and perineurial cells (Fite-Faraco, ×400).

Histopathological examination is a central component for confirming the diagnosis of leprosy and contributes decisively to classification within the disease’s immunopathological spectrum. Unequivocal demonstration of AFB within cutaneous nerves ‒ in Schwann cells or the perineurium ‒ is diagnostic and distinguishes leprosy from other mycobacterioses (Fig. 1A‒B).^5^ When morphology is equivocal, PCR can complement the assessment.

Although some services report bacillary burden qualitatively (scant, moderate, abundant), regardless of the quantification method, the topographic distribution of AFB should be documented (nerves, macrophages, endothelium, skin appendages).^6^ When quantification is undertaken, the Bacillary Index of Granuloma (BIG) on skin biopsy can reveal bacilli even when slit-skin smears are negative, and may upstage a subset of clinically PB patients to MB ‒ thereby preventing undertreatment.^7^

**Fig. 2:**
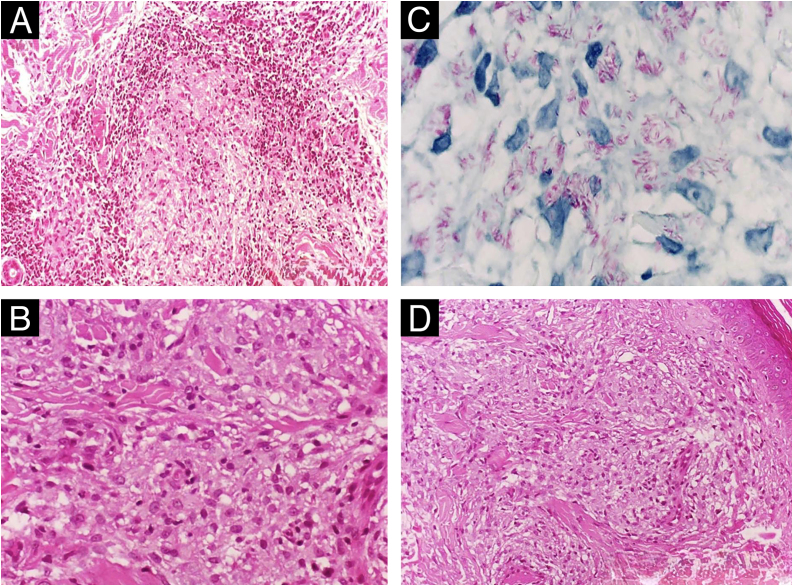
Histopathologic spectrum of leprosy. (A) Tuberculoid leprosy: well-formed epithelioid granulomas with a peripheral lymphocytic halo tracking neurovascular bundles in the dermis and extending into the subcutis (Hematoxylin & eosin, ×200; inset ×40). (B) Lepromatous leprosy: confluent infiltrate of macrophages with abundant vacuolated (“foamy”) cytoplasm (Hematoxylin & eosin, ×400). (C) Lepromatous leprosy: Fite–Faraco highlights numerous bacilli within macrophage cytoplasm (×1000). (D) Lepromatous leprosy: flattened epidermis separated from a macrophage-rich infiltrate by a subepidermal band of collagen (“grenz zone”) (Hematoxylin & eosin, ×100).

The histological patterns of leprosy span a characteristic immunopathological spectrum. At the tuberculoid pole, there are well-formed epithelioid granulomas contiguous with the epidermis, with epidermal thinning, epithelioid histiocytes, and Langhans-type giant cells, plus a dense peripheral infiltrate of CD4+ T-lymphocytes (Fig. 2A).^8^ Small perigranulomatous aggregates of B-lymphocytes (CD20+) may also be present, with subpopulations recently characterized.^9^ Bacilli are rare or absent, reflecting a robust cell-mediated immune response, whereas neural destruction can be pronounced.^10^

At the lepromatous (Virchowian) pole, diffuse infiltrates of foamy macrophages (Virchow cells) (Fig. 2B) packed with bacilli predominate, forming globi (Fig. 2C), without organized granulomas (Fig. 2D). A subepidermal grenz zone ‒ a narrow band of spared superficial dermis — separates the epidermis from the dermal infiltrate.^11^, ^12^ Nerves show bacillary invasion with minimal inflammatory response, developing onion-bulb perineural thickening due to perineurial hyperplasia and fibrosis secondary to chronic neural injury.^13^

Between the poles, the borderline forms display intermediate features, with granuloma organization inversely proportional to bacillary load.^10^ Along this progression: in borderline tuberculoid (BT), partially organized granulomas with scant bacilli; in borderline-borderline (BB), poorly defined granulomas with a moderate bacillary load; and in borderline lepromatous (BL), a predominance of histiocytes with numerous bacilli but residual, rudimentary granulomatous foci.^8^ Intralesional heterogeneity is characteristic, with variation in granuloma organization and bacillary density even within a single biopsy.

The indeterminate form represents the earliest stage of infection, characterized by a nonspecific lymphocytic infiltrate with perivascular, perineural, and periadnexal distribution, without well-formed granulomas.^14^ The paucity of bacilli at this stage limits the sensitivity of conventional methods; confirmation requires demonstrating intraneural AFB or molecular detection by PCR, when available.^15^ Clinically, it presents as a hypopigmented macule with subtle hypoesthesia and localized sudomotor involvement.^8^

The histoid variant is a peculiar morphological expression within the lepromatous spectrum, characterized by a proliferation of spindle-shaped histiocytes arranged in interlacing fascicles, a hyalinized stroma, exceptionally high bacillary density, and, paradoxically, few globi, with preservation of the characteristic grenz zone. Although historically associated with relapse after dapsone monotherapy, it is now recognized both in relapsed cases and in treatment-naïve patients managed with multidrug therapy. Given its histological resemblance to spindle-cell neoplasms on hematoxylin-eosin sections, Fite-Faraco staining is mandatory for diagnostic confirmation.^11^, ^12^, ^16^

**Fig. 3:**
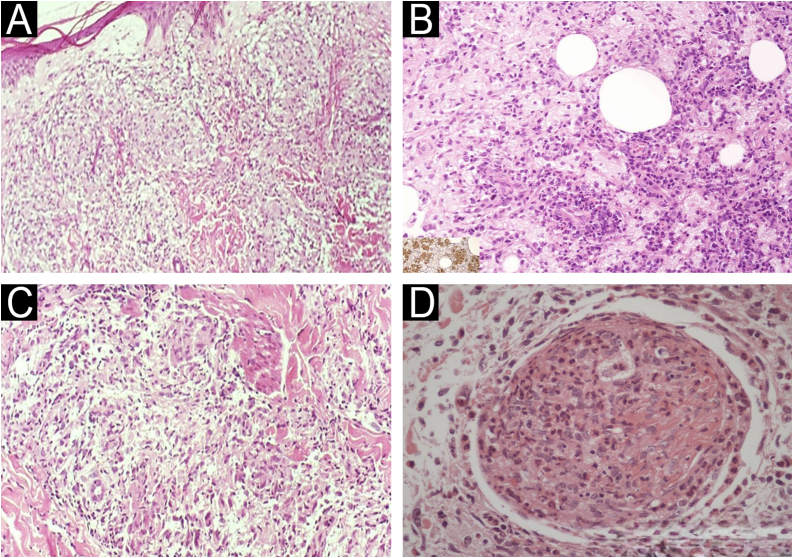
Leprosy reactions: type 1 vs. type 2. (A) Type 1 reaction: dermal edema separating components of the inflammatory infiltrate (Hematoxylin & eosin, ×40). (B) Type 1 reaction: epithelioid granuloma with edema-related dissociation of inflammatory cells (Hematoxylin & eosin, ×100). (C) Type 2 reaction: neutrophil-rich exudation within a subcutaneous fat lobule on a macrophage-rich background; inset, anti-BCG immunohistochemistry highlights mycobacterial antigen within macrophage cytoplasm (Hematoxylin & eosin for main panel, ×200; inset ×200). (D) Type 2 reaction: subcutaneous nerve permeated by neutrophilic exudate (Hematoxylin & eosin, ×200).

Acute reactional episodes can superimpose on the baseline architecture of the spectrum and have distinct morphological signatures. In type 1 reaction, one observes interstitial edema, intensification of the granulomatous response with increased epithelioid cells and multinucleated giant cells (Fig. 3A,B), and occasionally perineural or focal neural fibrinoid necrosis; these changes may be subtle or even absent, requiring clinicopathologic correlation.^17^, ^18^, ^19^ Rarely, severe reactions develop caseous necrosis, sometimes centered on dermal nerve twigs (segmental necrotizing granulomatous neuritis), producing nodular or abscess-like foci. These lesions can mimic cutaneous tuberculosis or, through their caseating morphology, tertiary syphilis. Intraneural/perineural AFB favor leprosy; AFB centered within necrosis without neural tropism suggest tuberculosis; species-specific PCR can confirm the etiology.^20^

In type 2 reaction, there is an acute neutrophilic infiltrate superimposed on a pre-existing lepromatous or borderline-lepromatous pattern (Fig. 3C), most conspicuous within the first 24–72 hours. Immune-complex deposition may produce lobular panniculitis, leukocytoclastic vasculitis, or acute neuritis (Fig. 3D); in delayed biopsies or after corticosteroid therapy, the neutrophilic component may be absent, hindering histologic recognition.^21^, ^22^, ^23^

A rare complication associated with untreated diffuse lepromatous leprosy is Lucio phenomenon, histologically characterized by necrotizing vasculopathy with heavy bacillary invasion of the endothelium, endothelial proliferation, and thrombosis of medium-sized dermal vessels, typically without a prominent neutrophilic infiltrate in the early phases. Clinically, it presents with painful, irregular cutaneous ulcers resulting from tissue infarction secondary to vascular occlusion.^24^, ^25^

Technical considerations are essential for accurate diagnosis across all forms. To maximize diagnostic yield, the skin biopsy should be taken from the actively infiltrated edge of the most recent lesion ‒ avoiding the atrophic center or areas of spontaneous regression ‒ and should include deep dermis and subcutaneous tissue, where nerves and adnexal structures concentrate bacilli.^26^ Absence of bacilli on Fite-Faraco staining does not exclude leprosy, particularly in tuberculoid forms in which bacillary paucity is characteristic. When a skin biopsy shows perineural inflammation even without demonstration of AFB, this finding strongly supports the diagnosis in a compatible clinical context.^27^

When the initial Fite-Faraco stain is negative in a clinically suggestive case, additional serial sections with meticulous examination of perineural areas can reveal scant bacilli initially missed, avoiding unnecessary re-biopsies. Although nonspecific, anti-BCG immunostaining can be a valuable adjunct: perineural positivity demonstrates mycobacterial antigens and guides a targeted review of the Fite-Faraco stain, increasing detection in paucibacillary cases.^28^

**Fig. 4:**
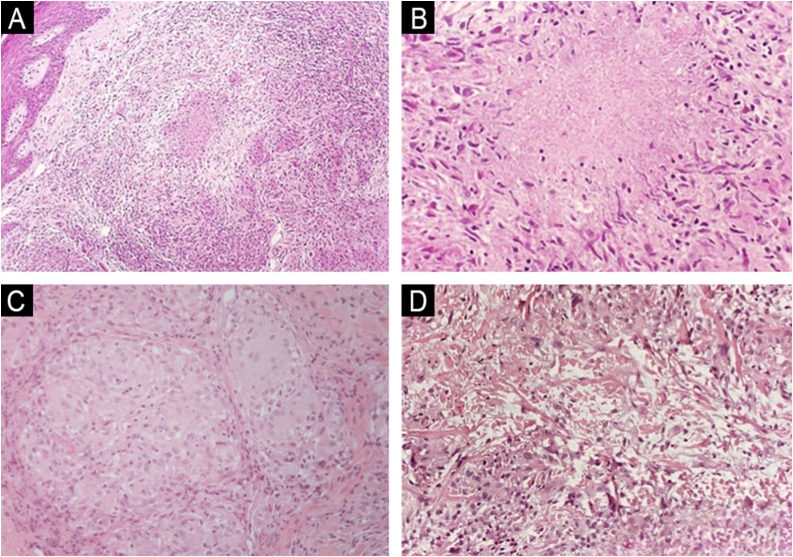
Differential diagnosis of granulomatous dermatoses. (A) Cutaneous tuberculosis: epithelioid granulomas, sometimes confluent, occupying the reticular dermis (Hematoxylin & eosin, ×100). (B) Cutaneous tuberculosis: fibrinoid necrosis within granulomas (Hematoxylin & eosin, ×200). (C) Sarcoidosis: compact (“naked”) epithelioid granulomas with minimal peripheral inflammation (Hematoxylin & eosin, ×200). (D) Granuloma annulare: palisading histiocytes surrounding foci of altered collagen; inset, Alcian blue highlights mucin within areas of collagen degeneration (Hematoxylin & eosin for main panel, ×200; inset ×200).

Key diagnostic pitfalls include: confusing tuberculoid leprosy with sarcoidosis by failing to recognize subtle perineural inflammation; misdiagnosing lepromatous leprosy as histiocytosis based solely on hematoxylin-eosin without performing Fite-Faraco staining; and overinterpreting nonspecific perineural infiltrates as leprosy in the absence of AFB or adequate clinicopathologic correlation. Major differential diagnoses include cutaneous tuberculosis (central caseation without neuritis) (Fig. 4A,B), sarcoidosis (“naked” granulomas sparing nerves) (Fig. 4C), granuloma annulare (central collagen degeneration) (Fig. 4D), and, in immunosuppressed patients, atypical mycobacteria such as *M. haemophilum*.^29^, ^30^, ^31^

Beyond the cutaneous findings described above, the neural component warrants dedicated assessment. At the tuberculoid pole, the intense granulomatous response leads to substantial neural destruction; at the lepromatous pole, massive bacillary invasion paradoxically preserves overall nerve architecture despite the high bacillary load.^32^ A perineural infiltrate is a sentinel finding and should prompt Fite-Faraco staining whenever leprosy is suspected. Importantly, in some patients, nerve histopathology does not mirror skin findings (e.g., paucibacillary skin versus a more bacilliferous, less organized intraneural infiltrate); therefore, classification must integrate skin and nerve with the clinical picture to avoid mislabeling relapse or underestimating inflammatory activity.^33^, ^34^

In primary neural leprosy, when indicated, nerve biopsy should be interpreted in conjunction with clinical data, nerve conduction studies, ultrasonography, and molecular testing to secure the diagnosis.^35^ When biopsy is not feasible, Fine-Needle Aspiration (FNA) of the affected nerve trunk ‒ ideally ultrasound-guided to target thickened/hypervascular segments ‒ can assist, allowing cytology, Fite-Faraco staining, and PCR for *M. leprae* from the same material.^36^, ^37^

When neural sampling is required, sensory nerves or their branches are preferred ‒ sural (first choice), superficial radial at the wrist, or the dorsal cutaneous branch of the ulnar nerve ‒ to minimize the risk of permanent motor deficit; when only mixed nerves are affected, biopsy may be necessary despite the risk of sequelae.^38^ Processing should include hematoxylin-eosin and Fite–Faraco, with evaluation of overall architecture, inflammatory infiltrates, and perineural changes. Key differentials for the neural form include vasculitis (vascular fibrinoid necrosis), chronic inflammatory demyelinating polyneuropathy (prominent segmental demyelination), and neuropathies due to atypical mycobacteria in immunosuppressed patients (lacking the characteristic neural tropism of *M. leprae*).

### Molecular detection of *M. leprae*

Polymerase Chain Reaction (PCR) detects *M. leprae*-specific DNA sequences with higher sensitivity than bacilloscopy or Fite-Faraco staining on tissue. Main indications include clinically suspected leprosy with negative bacilloscopy ‒ especially in paucibacillary forms ‒ diagnostic confirmation in primary neural leprosy, and differentiation between late reactional episodes and relapse. Sensitivity varies by sample type and clinical form. In skin biopsies, the sample of choice, sensitivity reaches 90%–100% in multibacillary cases and 34%–80% in paucibacillary cases, reflecting a direct correlation with bacillary load.^39^, ^40^, ^41^

For optimal sampling, biopsy the active edge of the most recent lesion or the one with the most evident sensory change. When histopathology and PCR are both needed, collect two distinct samples: one fixed in formalin for histopathology and another unfixed, kept refrigerated, for molecular testing. Previously paraffin-embedded tissue can be used, although with reduced sensitivity due to DNA fragmentation during processing.^42^, ^43^, ^44^

Result interpretation requires understanding the inherent limitations of PCR. Since bacterial DNA remains stable for extended periods, positive results may persist years after successful treatment, without indicating therapeutic failure or active disease. This persistence of residual genetic material is particularly relevant when evaluating previously treated patients, where an isolated positive result without compatible clinical findings neither establishes relapse nor justifies restarting multidrug therapy. Conversely, a negative result does not exclude the diagnosis, especially in paucibacillary forms where bacillary numbers often fall below the assay’s detection threshold.^45^, ^46^ In nasal swabs ‒ particularly in endemic areas ‒ positivity may reflect transient colonization and, without a compatible clinical picture, does not establish active disease.^40^, ^47^, ^48^ Given these limitations, quantitative reading of the Cycle threshold (Ct) in real-time PCR, when available, can assist in estimating bacillary load.

**Fig. 5:**
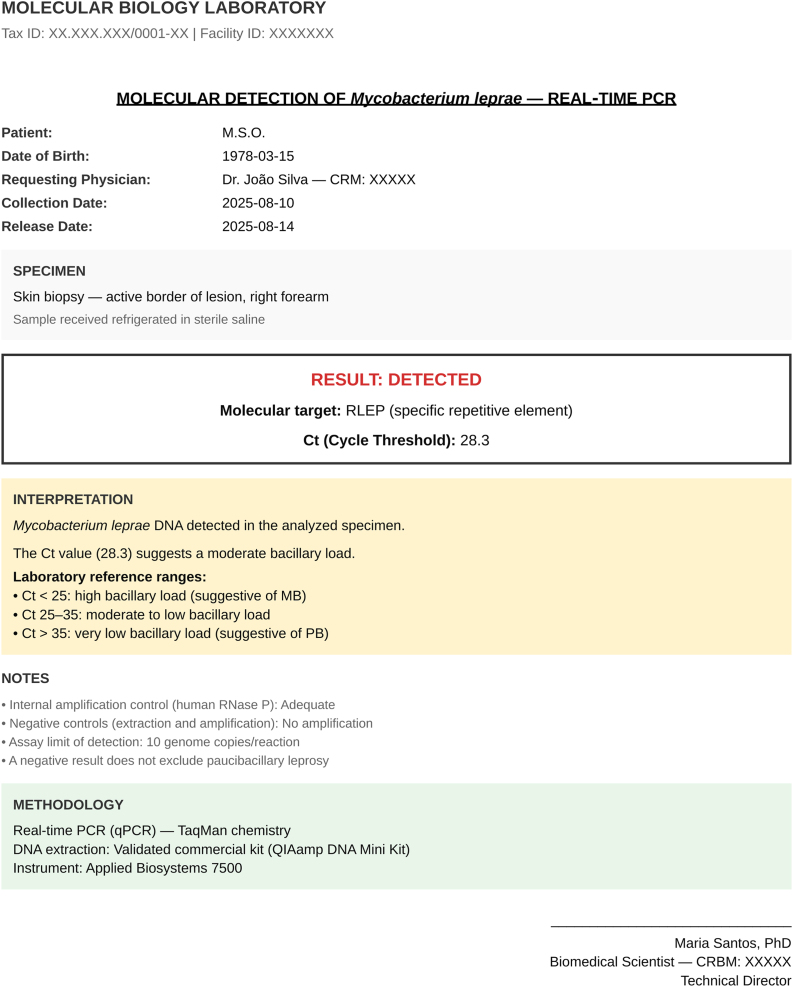
Example of a real-time quantitative PCR report for *Mycobacterium leprae* (RLEP target) from a biopsy of the active edge of a lesion, showing a positive result (Ct 28.3). Ct is inversely proportional to bacillary load; in this laboratory, values < 25 indicate high load (multibacillary forms), 25–35 moderate load, and > 35 low load (paucibacillary forms). Valid internal and negative controls confirm assay quality.

The Ct value ‒ the number of cycles required to detect DNA ‒ is inversely proportional to bacillary load and provides a useful semiquantitative estimate: lower values indicate greater amounts of bacterial DNA. Although cutoffs vary across laboratories, Ct values < 25 typically indicate high load (multibacillary forms), 25–35 moderate load, and > 35 low load (paucibacillary).^49^ In the example in Fig. 5, a positive result with Ct 28.3 corresponds to an intermediate load. Importantly, PCR detects DNA fragments and cannot distinguish viable from non-viable bacilli; therefore, a single Ct value does not differentiate a reactional episode ‒ an immuno-inflammatory phenomenon without a true increase in bacillary burden ‒ from relapse, which reflects resumption of replication.

In suspected primary neural leprosy, a minimally invasive approach can optimize diagnostic yield before considering nerve biopsy. Real-time PCR on slit-skin smears from clinically unaffected “cold sites” (earlobes, elbows, knees) frequently detects subclinical cutaneous involvement. In a Brazilian cohort of primary neural leprosy, slit-skin PCR demonstrated 78.6% positivity and was the sole positive test in 17.1% of cases, whereas nerve PCR reached 60.8% positivity, and nerve bacilloscopy was rarely positive ‒ findings that support a stepwise diagnostic algorithm reserving nerve biopsy for cases remaining negative or inconclusive after non-invasive testing.^50^ Independent series using the multicopy RLEP target corroborate high sensitivity and specificity of slit-skin PCR across clinical forms, with practical advantages including filter-paper storage for resource-limited settings.^39^, ^51^ When these non-invasive approaches fail to establish a diagnosis, molecular analysis of a nerve fragment can be pursued.

In primary neural leprosy, molecular analysis of a nerve fragment shows positivity in about 60% of cases,^50^, ^52^ even when bacilloscopy is negative. When PCR positivity in nerve coincides with topographically compatible electroneuromyographic abnormalities, these findings together support the diagnosis in this clinically challenging scenario.^53^

### Serologic testing (anti-PGL-1)

Leprosy diagnosis remains essentially clinical, grounded in the recognition of cutaneous lesions and neural abnormalities. Detection of IgM anti-PGL-1 ‒ antibodies against phenolic glycolipid-1, a species-specific cell-wall component of *M. leprae* ‒ is an auxiliary tool with limited application whose interpretation requires careful attention to indications and limitations. A rapid immunochromatographic test performed on capillary blood (finger-prick) yields a visual readout in 10–15 minutes and can be carried out at the point of care.

Regarding performance, a meta-analysis of anti-PGL-1 rapid tests reported 92% sensitivity and 93% specificity in multibacillary disease, and 36% sensitivity with 94% specificity in paucibacillary forms.^54^ Sensitivity varies widely by clinical form and bacillary load: lepromatous (Virchowian) forms exceed 95% positivity; borderline forms range from 70%–85%; whereas tuberculoid and indeterminate forms show only 15%–40%.^55^, ^56^ This pattern reflects immunologic polarization: multibacillary patients mount a strong humoral response, while paucibacillary disease is dominated by a Th1 (CD4^+^) cellular response and typically lacks detectable antibodies.

In practice, the test is most useful for evaluating household and social contacts. In prospective studies, seropositive contacts had a relative risk of 3.54 (95% IC 2.21–5.67) for developing leprosy, supporting targeted surveillance.^57^, ^58^, ^59^ Anti-PGL-1 markers have also been associated with risk of neural involvement, underscoring the importance of neurologic follow-up.^60^ Based on current guidelines, contacts with positive serology but no clinical manifestations require annual surveillance for five years.^2^ In contacts with inconclusive clinical findings, a reactive result heightens suspicion and guides further investigation.

Misinterpretation of serology is a frequent source of diagnostic error. In endemic areas, 15%–20% of healthy individuals show IgM anti-PGL-1 due to prior exposure without active disease.^61^ The risk of misdiagnosis increases in the setting of non-leprosy hypopigmented lesions (pityriasis, post-inflammatory hypopigmentation, vitiligo), where concurrent seropositivity can prompt unnecessary treatment. In the absence of sensory impairment, localized dysautonomia, or nerve thickening, isolated seropositivity does not support the diagnosis. Given these limitations, the anti-PGL-1 test should not be used for population screening or as a standalone diagnostic criterion, remaining strictly complementary to clinical-epidemiological assessment.^13^

Primary neural leprosy exemplifies a critical limitation of serology. This form ‒ peripheral neuropathy without cutaneous lesions ‒ consistently yields negative serology due to the predominance of cellular immunity. A negative result in such cases can inappropriately exclude the diagnosis, delaying treatment of the clinical form most often associated with permanent physical disability. Similarly, in indeterminate and tuberculoid forms, where early detection is crucial to prevent sequelae, sensitivity below 30% renders the test of limited value. Thus, a negative result does not exclude leprosy when the clinical picture is suggestive.^60^

## Historical note — Lepromin (Mitsuda) test

The lepromin (Mitsuda) skin test historically indexed cell-mediated immunity to *M. leprae*. After intradermal inoculation of killed bacilli, an early Fernández reaction at 24–48 h and a late Mitsuda nodule at 21–28 days reflect a Th1-dominant response (IL-12/IFN-γ/TNF-α) ‒ typically positive in tuberculoid/borderline-tuberculoid disease. Conversely, lepromatous/borderline-lepromatous disease shows Th2/regulatory skewing (IL-4/IL-5/IL-10) with lepromin anergy. Owing to non-standardized antigen preparations and the lack of diagnostic/therapeutic utility at the individual level, the test is no longer recommended in routine clinical practice, with current use largely confined to epidemiologic and immunologic studies.^62^, ^63^

## Autonomic tests

Autonomic dysfunction is an early and consistent feature of leprosy neuropathy, primarily affecting unmyelinated C-fibers that mediate sudomotor and vasomotor responses. At the bedside, two complementary tests interrogate distinct components: the histamine test maps the C-fiber-mediated axon reflex (vasomotor), whereas the pilocarpine test probes sudomotor function through direct sweat-gland stimulation.^2^

### Histamine test

The histamine test assesses the integrity of cutaneous autonomic nerve fibers via the C-fiber-mediated axon reflex, allowing direct mapping of neural dysfunction within the suspect lesion. Its main utility is in indeterminate forms with subtle sensory changes ‒ particularly in lower Fitzpatrick phototypes (I–III) ‒ and in children or patients who cannot reliably report sensory changes during Semmes-Weinstein monofilament testing. Autonomic involvement manifests as attenuation or absence of the peripheral flare (axon-reflex erythema), a component of Lewis’s triple response.^64^

The test can be performed using two modalities: exogenous histamine and endogenous histamine (dermographism). The exogenous test uses an intradermal injection of 0.1 mL histamine 1:1000 at the lesion border and at a contralateral control site. The expected sequence, known as Lewis’s triple response, comprises primary erythema at 15–30 seconds, a peripheral reflex erythema (flare) at 30–60 seconds driven by neuropeptide-mediated vasodilation, and a 3–4 mm wheal at 2–3 minutes due to plasma extravasation.

The 10-minute reading compares the diameter of the peripheral flare between the lesion and the control. A complete response displays both wheal and flare at both sites; an incomplete response reveals wheal formation with absent or attenuated flare within the lesion, indicating local autonomic dysfunction. In indeterminate leprosy, an incomplete response at the active edge increases diagnostic probability and identifies the optimal biopsy site.^64^

**Fig. 6:**
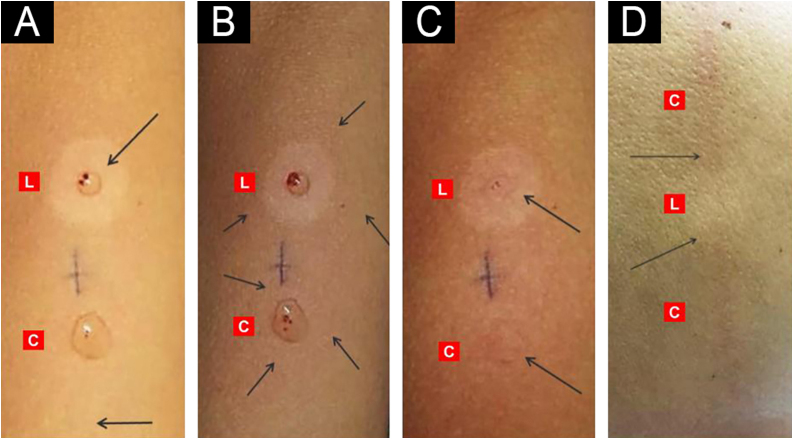
Exogenous (A–C) and endogenous (D) histamine tests. (A) Erythema confined to the puncture site at 15–20 seconds, present in both Lesion (L) and Control (C) skin. (B) Axon-reflex erythema (flare) at 30–60 seconds, present only in Control (C) skin and abolished in the Lesion (L). (C) Wheal formation at 2–3 minutes, visible in both areas (L and C). (D) Dermographism after a linear mechanical stimulus, observed in Control (C) skin and absent within the Lesion (L).

Dermographism evaluates endogenous histamine released from mast cells after mechanical stimulation. A line is drawn with a blunt object (e.g., a pen cap) using firm, continuous pressure from proximal normal skin across the suspect lesion to distal normal skin. In leprosy, the flare is reduced or absent within the lesion. While simpler to perform and requiring no pharmacological preparation, dermographism has lower diagnostic sensitivity than exogenous histamine testing. Fig. 6 documents the temporal sequence of the tests and abolition of the flare within the lesion, with preservation at the control site.

Limitations include non-specificity ‒ an incomplete response also occurs in other neuropathies and in scarred areas. *Nevus anemicus* is an important diagnostic pitfall: it yields an “incomplete response” due to vascular hypersensitivity to catecholamines rather than denervation. It is distinguished by a congenital history, blanching on diascopy, and absence of sensory abnormalities.

### Pilocarpine sweat test

The pilocarpine test complements histamine by directly assessing sudomotor output. After painting the skin with tincture of iodine at the lesion border and a contralateral control site, 0.1–0.2 mL of pilocarpine (0.5%–1%) is injected intradermally, and the area is dusted with starch. Normal sweating produces dark discoloration (iodine-starch reaction); reduced or absent discoloration within the lesion compared with the control indicates sudomotor dysfunction. This objective mapping is particularly helpful in patients in whom visualizing the histamine flare is difficult (e.g., darker phototypes) and in children.^2^

### Monofilament testing (Semmes-Weinstein)

Semmes-Weinstein monofilament testing is the most useful point-of-care assessment for detecting and monitoring peripheral nerve function in leprosy. Although thermal tests ‒ especially heat-detection thresholds ‒ may reveal abnormalities earlier, monofilaments offer practical advantages by quantifying tactile thresholds in a standardized manner and enabling objective longitudinal documentation.^65^, ^66^

Testing should be performed at diagnosis and repeated periodically, particularly during the first year, when neurological deterioration and reactional episodes are more frequent, even under multidrug therapy.^67^, ^68^

A standardized set of five monofilaments is applied perpendicularly until it bends, with brief contact, while the patient's eyes are closed ‒ indicating perception. The smallest filament perceived at each point is recorded. Test the following territories bilaterally at standardized sites: ulnar nerve (fifth digit pulp and hypothenar eminence), median nerve (thumb pulp and thenar eminence), and radial cutaneous nerve in the upper limb; posterior tibial nerve (plantar pulps) and sural nerve (lateral foot border) in the lower limb.^69^

Mapping may reveal a patchy or “island” pattern ‒ hypoesthetic areas interspersed with preserved points ‒ reflecting irregular involvement of cutaneous fascicles with a predilection for cooler areas. This contrasts with the symmetric glove-and-stocking distribution typical of diabetic neuropathy and aids in differential diagnosis.^65^, ^70^, ^71^

For longitudinal assessment, small but consistent changes guide clinical management and allow detection of “silent” neuropathy. Clinically meaningful worsening is defined as the need for heavier filaments at two or more points within the same previously stable territory, or newly absent perception of light pressures at a site previously normal; repeating the test over a short interval is recommended to confirm the trend.^66^, ^72^

Once worsening is confirmed, integrate these findings with the neurological examination (assessing thickening and tenderness of the corresponding nerve trunk) and, when indicated, complement with ultrasonography to document intra- or perineural thickening and vascularity, as well as nerve-conduction studies to characterize demyelinating versus axonal patterns.^30^ In parallel, monitor voluntary strength in target muscles ‒ interossei (ulnar) and abductor pollicis brevis (median) ‒ since loss of strength often accompanies sensory deficit and precedes disability.^69^, ^73^, ^74^

**Fig. 7:**
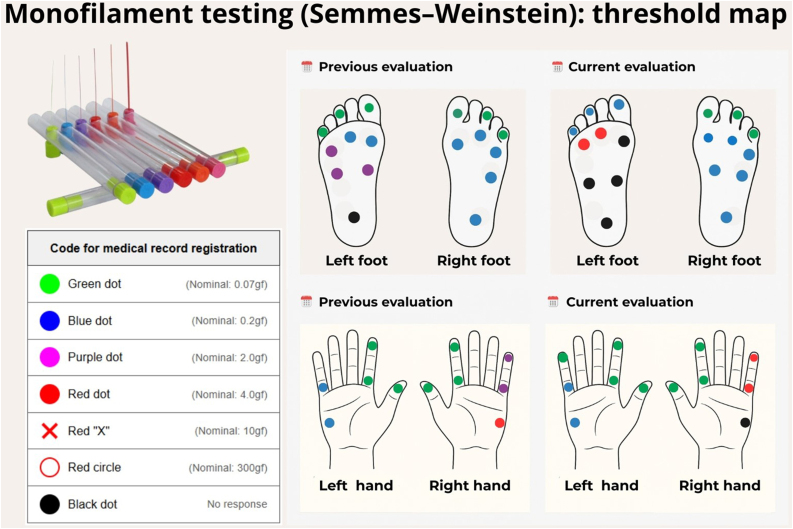
Semmes-Weinstein monofilament testing: bilateral mapping of tactile thresholds in the hands and feet. Each dot records the smallest filament perceived at the tested site; colors follow the key shown. Comparing the “prior assessment” with the “current assessment” allows recognition of clinically meaningful worsening ‒ the need for heavier filaments at ≥ 2 points within the same territory, or the emergence of non-responsive areas ‒ consistent with progressive neuritis in the right ulnar and left posterior tibial nerves.

As illustrated in Fig. 7, bilateral mapping and longitudinal comparison can document clinically meaningful worsening ‒ shifts toward heavier filaments at multiple points and the appearance of non-responsive sites ‒ consistent with progressive neuritis in the right ulnar and left posterior tibial nerves.

### Nerve conduction studies and electromyography

Electrodiagnostic studies ‒ comprising Nerve Conduction Studies (NCS) and needle Electromyography (EMG) ‒ specifically evaluate peripheral nervous system function by recording Sensory Nerve Action Potentials (SNAPs), Compound Muscle Action Potentials (CMAPs), and sensory and motor conduction velocities along spinal and cranial peripheral nerves.^75^ In leprosy, these studies detect abnormalities approximately 12-weeks before they become clinically evident or detectable by monofilament testing.^65^

Leprosy neuropathy (LN) typically presents as an asymmetric multiple mononeuropathy, requiring assessment of multiple nerves. Its protracted, indolent course warrants planned follow-up according to the disease phase ‒ initial, reactional, and late ‒ roughly following a biphasic pattern. Demyelinating changes arise early, manifesting as CMAP temporal dispersion, reduced conduction velocity, and prolonged latencies, particularly at common entrapment sites where neural vulnerability is greater. As the disease progresses, progressive inflammation and fibrosis lead to axonal loss, identified by progressive reductions in SNAP and CMAP amplitudes.^71^, ^76^

During reactional episodes, the electrodiagnostic pattern shifts: acute conduction blocks, more frequent in Type 2 Reactions (T2R), and subacute temporal dispersion in Type 1 Reactions (T1R) ‒ both potentially reversible with immunosuppressive therapy (corticosteroids or agents such as cyclosporine and azathioprine).^35^ Established axonal loss has limited potential for recovery. These findings are characteristic of inflammatory demyelinating neuropathies, including Guillain-Barré syndrome and chronic inflammatory demyelinating polyradiculoneuropathy (CIDP), which should be considered in the differential diagnosis of LN.^77^

**Fig. 8:**
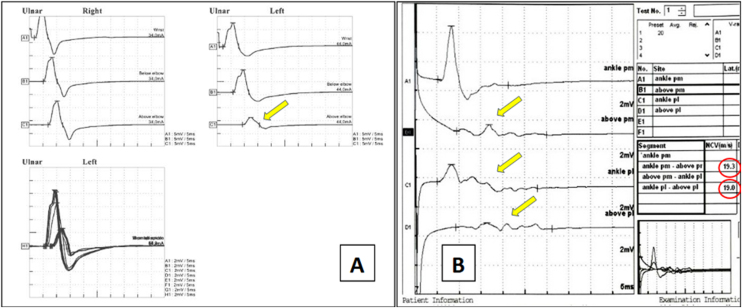
Electrodiagnostic findings in demyelinating neuropathy. (A) Bilateral ulnar motor conduction. Left: Normal right ulnar nerve. Right: Left ulnar nerve with partial conduction block (>50% CMAP reduction above elbow, yellow arrow). (B) Right tibial nerve at ankle. A1/B1: Medial plantar; C1/D1: Lateral plantar nerve. Progressive temporal dispersion evident in B1/D1 (red arrows). Reduced velocities across the tarsal tunnel (19.3‒19.0 m/s, circled). Conduction block and temporal dispersion characterize segmental demyelination.

Needle EMG complements NCS by documenting active denervation through abnormal spontaneous activity (fibrillations and positive sharp waves) and by characterizing reinnervation via motor-unit potential analysis. In leprosy, muscles innervated by the most frequently affected nerves ‒ tibial, common fibular, ulnar, and median (in decreasing order) ‒ may show acute abnormalities not captured by routine NCS. These findings corroborate axonal involvement and inform prognosis.^76^, ^78^ Fig. 8 illustrates electrodiagnostic changes characteristic of leprosy neuropathy.

Asymmetric sensory involvement is the predominant finding in electrodiagnostic reports of leprosy and typically precedes motor abnormalities. Exclusive motor involvement without sensory changes suggests alternative diagnoses ‒ such as motor neuron disease, radiculopathies, or myopathies ‒ although leprosy cannot be entirely excluded in specific epidemiological contexts.^65^ The main indication for NCS/EMG is suspected primary neural leprosy, characterized by sensory loss without cutaneous lesions; in this setting, testing helps confirm neural involvement and distinguish leprosy from other neuropathies.^79^

Interpretation requires recognizing the asymmetric multiple mononeuropathy pattern typical of leprosy. This pattern contrasts with diabetic neuropathy, which evolves with a symmetric distal-proximal distribution; focal entrapment syndromes, which are confined to the compression site; and vasculitic neuropathies, which present acutely with severe deficits and an axonal pattern from the outset. The asymmetric involvement of multiple nerves, with sensory predominance and an insidious demyelinating course, strongly suggests leprosy.^71^

**Fig. 9:**
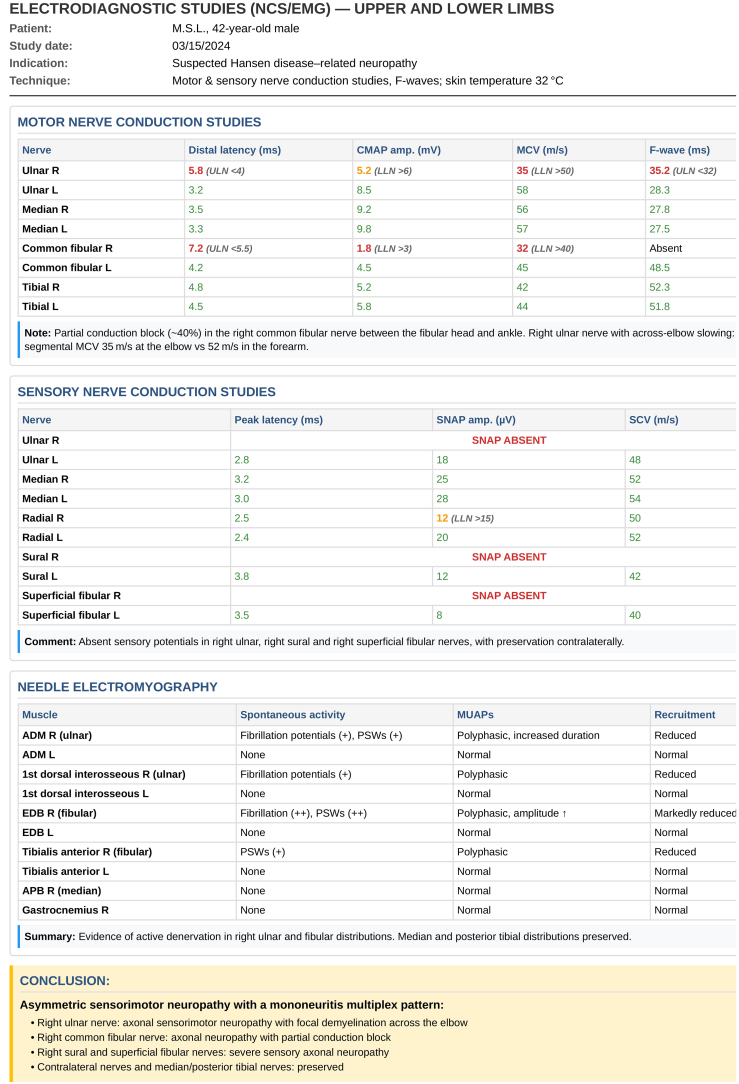
Example electrodiagnostic report in leprosy. Ulnar nerve with reduced motor conduction velocity (35 m/s; normal > 50 m/s) and absent sensory response; common fibular nerve with partial conduction block at the fibular head; median and tibial nerves preserved. Pattern consistent with asymmetric multiple mononeuropathy.

In reactional neuritis, whether clinically overt or silent, serial studies every 30–60 days can document progressive deterioration. Early changes include slowed conduction velocities and prolonged latencies, indicating active demyelination. With persistent inflammation, reductions in CMAP amplitudes emerge, signaling secondary axonal loss and a less favorable prognosis for recovery. NCS/EMG demonstrates superiority over other methods by characterizing in real time the pathophysiologic evolution of leprosy neuropathy.^80^, ^81^ Fig. 9 illustrates a typical electrodiagnostic report in leprosy, demonstrating the hallmark asymmetric multiple mononeuropathy pattern.

NCS/EMG primarily assess large, myelinated fibers; therefore, they have low sensitivity for isolated small-fiber involvement affecting thermal and pain sensation, potentially yielding normal results despite symptoms. Conversely, acute or subacute demyelination is readily detected on motor conduction as prolonged latencies, slowed velocity, conduction block, and temporal dispersion.^70^, ^73^ Sensory conduction tends to parallel Semmes-Weinstein monofilament testing (both assess large fibers), although monofilament testing is less sensitive than NCS/EMG for early detection of abnormalities.^65^, ^69^

### Ultrasonography

Peripheral nerve ultrasonography is a noninvasive method that provides detailed morphological evaluation of neural structures using high-frequency linear transducers (12–18 MHz). The examination offers objective measurements of nerve thickening and identifies signs of inflammatory activity with color Doppler, complementing clinical and electrodiagnostic evaluation in leprosy.^82^, ^83^

The main findings include increased Cross-Sectional Area (CSA), changes in internal texture, and intra- or perineural hypervascularization. Nerve thickening ‒ CSA exceeding normal limits ‒ is the most consistent finding; values > 10 mm^2^ are considered abnormal for the ulnar and median nerves, and > 15 mm^2^ for the common fibular nerve, although these reference ranges vary across populations.^84^

The normal fascicular architecture ‒ discrete fascicles within connective tissue ‒ becomes replaced by heterogeneous echogenicity reflecting edema, cellular infiltration, or fibrosis. During reactional episodes, color Doppler demonstrates increased intraneural vascularity ‒ an observation that correlates with active neuritis and may precede clinical manifestations.^73^, ^83^

Examination technique requires systematic bilateral scanning of the most frequently involved nerves. The ulnar nerve should be evaluated with particular attention to the cubital tunnel and proximal segment, where thickening is most pronounced. The median nerve is examined in the mid and distal thirds of the forearm, and the common fibular nerve at the fibular head. Bilateral assessment allows identification of asymmetries, a characteristic finding of leprosy-related multiple mononeuropathy.^83^

**Fig. 10:**
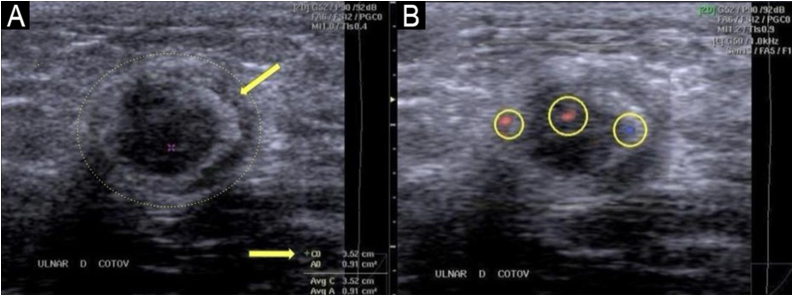
Ultrasonography of the ulnar nerve (transverse plane at the elbow). (A) B-mode imaging reveals marked thickening with a perimeter of 3.52 cm and an area of approximately 0.91 cm^2^ (≈91 mm^2^); for reference, normal is approximately 0.10 cm^2^ (≈10 mm^2^). Note epineurial thickening and a crescent-shaped hyperechoic halo (arrow). (B) Color Doppler at the same level shows three foci of peri-/intraneural flow (circles 1–3) within the Doppler box, consistent with inflammatory neuritis.

Documentation should include transverse CSA measurements, echotexture description, and Doppler assessment (81). A leprosy-typical pattern is defined by either (i) ≥ 2 thickened nerves exceeding reference limits, or (ii) side-to-side asymmetry (ΔCSA > 2.5 mm^2^; difference in CSA at the same site on right vs. left) combined with focal thickening along the same nerve (> 2.5 mm^2^ difference between proximal and distal sites); in a Brazilian multicentre series these criteria yielded ∼76% sensitivity and ∼88% specificity.^85^ Fig. 10 demonstrates typical ultrasonographic findings in leprosy.

Ultrasonography offers significant advantages over clinical palpation for detecting nerve thickening. The kappa coefficient, which measures agreement between methods [ranging from 0 (no agreement) to 1 (perfect agreement)], is only 0.30 between palpation and ultrasound, indicating low concordance. This shows that many nerves found to be thickened on ultrasound are not detected by palpation, and conversely, nerves considered thickened on palpation may measure normal on ultrasound.^82^

**Fig. 11:**
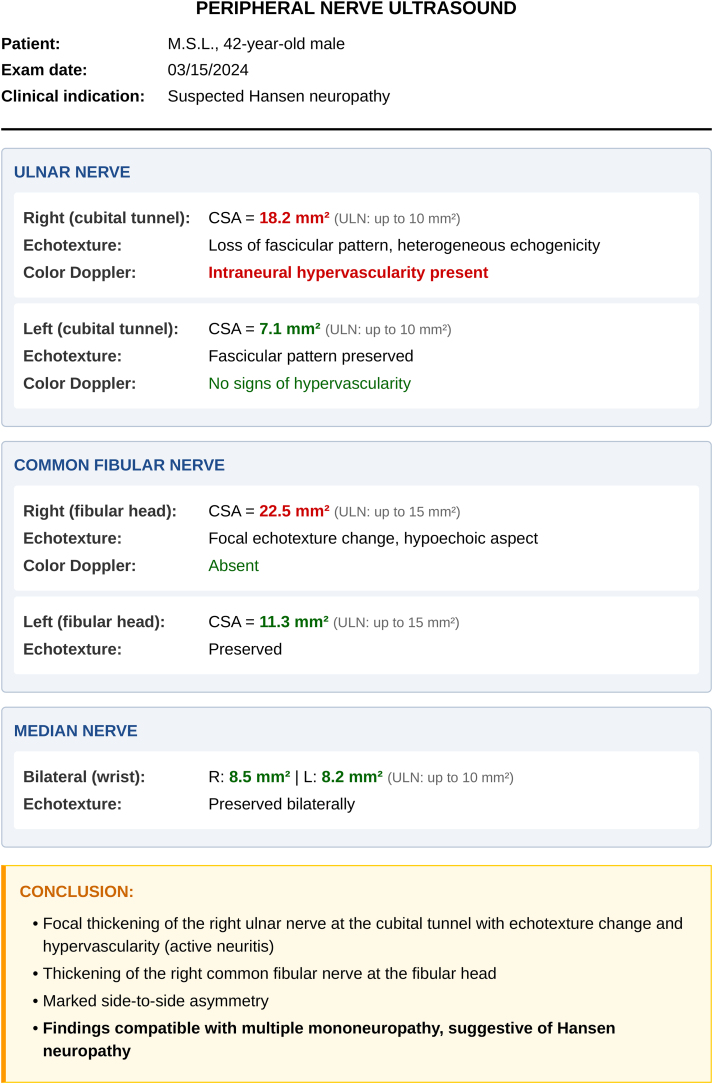
Example ultrasonographic report in leprosy. Focal thickening of the right ulnar nerve at the cubital tunnel (CSA 18.2 mm^2^; normal < 10 mm^2^), with loss of the fascicular pattern, heterogeneous echogenicity, and increased intraneural flow on color Doppler ‒ findings indicative of active neuritis. There is also thickening of the right common fibular nerve (CSA 22.5 mm^2^) with focal echotextural change. Comparison with the contralateral sides shows marked asymmetry. These findings are consistent with an asymmetric multiple mononeuropathy of leprosy pattern.

In post-treatment follow-up, paradoxical changes may occur. Some patients show increased nerve thickening despite adequate multidrug therapy, whereas others show reductions. The increase may represent evolving Type 1 Reaction (T1R), requiring corticosteroids, or simply reflect residual inflammatory activity without clinical significance. Conversely, reduction does not necessarily indicate improvement and may represent atrophy secondary to irreversible axonal damage. This apparent contradiction reinforces that ultrasonographic findings alone are insufficient ‒ imaging must always be correlated with clinical manifestations such as nerve pain and worsening sensory or motor function.^83^ Fig. 11 provides an example of a typical ultrasonographic report of an ulnar nerve thickened by leprosy.

Compared with Nerve Conduction Studies/Electromyography (NCS/EMG), ultrasonography shows higher sensitivity (97.4% vs. 78.9%) but lower specificity (69.4% vs. 93.5%).^86^ For post-treatment follow-up, these data are relevant, since neurophysiological abnormalities detected by NCS/EMG are generally more specific than the morphological changes observed on ultrasound.

When planning a nerve biopsy, ultrasonography identifies segments with greater thickening and hypervascularization, guiding the biopsy to regions with a higher likelihood of diagnostic abnormalities. The method also assists in differentiating compressive neuropathies, which show focal thickening confined to the anatomical site of compression, in contrast to the broader, asymmetric pattern typical of leprosy.^82^, ^87^

However, nerve thickening is not specific to leprosy. Beyond focal entrapments at common sites (carpal tunnel, cubital tunnel), several conditions may present with nerve thickening and hypervascularization ‒ hereditary neuropathies such as Charcot-Marie-Tooth disease, HNPP, and familial amyloid polyneuropathy; inflammatory neuropathies including Guillain-Barré syndrome and multifocal motor neuropathy (and multifocal sensory-motor neuropathy); as well as neurofibromatosis and nerve-sheath tumors.^88^ Variability in reference values across populations and the absence of universal standardization also limit interpretation.^83^ Findings should therefore be interpreted in light of the regional prevalence of these conditions, avoiding precipitous diagnosis of leprosy.

### Integrated diagnostic approach

Proper interpretation of complementary tests is essential when evaluating patients who worsen during or after leprosy treatment. Three conditions frequently overlap clinically because they share similar manifestations: the appearance of new lesions, worsening of preexisting lesions, or progression of neural involvement. Therapeutic failure presents as persistence or progression of lesions despite regular treatment, with viable bacilli persisting. Late reactional episodes are characterized by the sudden reappearance of old lesions or emergence of new ones, without bacillary multiplication. Relapse presents with the gradual reappearance of typical leprosy lesions after a period of cure, indicating renewed bacterial multiplication.^2^, ^89^, ^90^, ^91^

The interval since treatment completion is a key orienting element for the differential diagnosis. Therapeutic failure manifests during active treatment, before the end of multidrug therapy. Late reactions occur predominantly in the first three years after treatment completion, a period in which immunologic phenomena remain frequent. After 3–5 years, the probability of relapse increases progressively, reflecting the time needed for renewed bacillary multiplication. While not absolute, this temporal distinction guides subsequent laboratory investigation.^2^, ^23^

In multibacillary cases, slit-skin smear microscopy provides useful parameters for differentiation. In therapeutic failure, uniformly stained bacilli persist despite ongoing treatment, with a Bacteriological Index (BI) that is unchanged or declines insufficiently. Reactional episodes show a stable or expectedly declining BI (≈1 log/year), with only fragmented or granular forms. In relapse, there is an increase in BI of ≥2-points compared with the BI at treatment completion at any site, with the presence of uniformly stained, solid bacilli. In paucibacillary cases with negative bacilloscopy, correlation with other parameters is required.^2^, ^92^

Histopathology adds significant diagnostic value. When therapeutic failure is suspected, a histological Bacillary Index (hBI) ≥3+ showed 73% sensitivity and 78% specificity for discrimination. The presence of foamy granulomas increases the likelihood of therapeutic failure by 7.36-fold, and persistent neural/perineural infiltrate together with a high hBI further supports this suspicion. Type 1 reactional episodes show intensification of the granulomatous response with interstitial edema and increased epithelioid and giant cells, but without an increase in bacillary load. In relapse, the characteristic pattern includes the reappearance of foamy macrophages with viable bacilli and reconstitution of the grenz zone.^2^, ^4^, ^18^

PCR provides semiquantitative information via the Cycle threshold (Ct), inversely proportional to bacillary load. Ct values < 25 suggest high load (active multibacillary forms), 25–35 moderate load, and > 35 low load or residual fragments. In therapeutic failure, PCR remains persistently positive with low Ct values, reflecting maintenance of bacillary burden despite treatment. Reactional episodes show high or undetectable Ct, consistent with only bacillary fragments. In relapse, PCR is positive with low Ct in association with solid bacilli and a rising BI. In paucibacillary cases with negative bacilloscopy, positive PCR three years after treatment completion, especially with low Ct, points toward relapse.^40^, ^41^

Anti-PGL-1 serology has complementary value but important limitations. Titers decline by approximately 50% per year during and after adequate treatment.^92^ Persistently high or rising titers after multidrug therapy suggest therapeutic failure or relapse. An ELISA index ≥3.95 shows 79% sensitivity but only 59% specificity for therapeutic failure.^92^ Serology alone does not determine management, as antibodies can persist for years after effective bacillary clearance.^93^

An integrated approach maximizes diagnostic accuracy. The combination of hBI ≥3+, anti-PGL-1 ≥ 3.95, and positive PCR with neural/perineural infiltrate provides a 95% predictive value for therapeutic failure.^4^ Joint analysis should consider: time since treatment completion; BI trajectory relative to baseline; bacillary morphology (solid vs. fragmented); histopathological pattern (foamy granulomas, neural infiltrate); PCR Ct-value; and trends in serologic titers.^40^, ^41^, ^92^

When therapeutic failure is suspected or confirmed, testing for drug resistance is mandatory. Targeted PCR assays can identify mutations in folP1 (dapsone), rpoB (rifampicin), and gyrA (fluoroquinolones), enabling individualized treatment. For reliable molecular testing, specimens from cases with BI ≥ 2+ yield ∼90% sensitivity, whereas lower BI values reduce sensitivity to ∼20%–40%.^94^ Brazilian surveillance shows a low overall prevalence of molecular resistance (∼1.4%), largely confined to relapse or suspected treatment failure, which supports focusing testing on these scenarios.^95^

This integrated framework enables reliable distinction among therapeutic failure due to drug resistance, late reaction, and relapse, underpinning appropriate therapeutic decisions for each specific clinical scenario. Table 1 summarizes the main laboratory parameters for distinguishing late reaction from relapse ‒ the two situations that most commonly generate diagnostic uncertainty during post-treatment follow-up.

**Table 1:** Laboratory parameters for differentiating late reaction from relapse in leprosy.

Differential diagnosis: late reaction vs relapse			
	Reaction	Relapse	Utility
Release from treatment (RFT)	0–3 years	> 3–5 years	High
Multibacillary (MB)			
Slit-skin smear (BI)	BI declining by ∼1 log/year; fragmented and/or granular bacilli	BI ≥ 2 points above the value at treatment completion and/or uniformly stained	High ‒ official criterion
Histopathology	Interstitial edema; intensified granulomatous response; increased epithelioid/giant cells	New foamy macrophages; reconstituted grenz zone; uniformly	Moderate ‒ macrophage-rich infiltrate does not persist for years
PCR	Positive with high Ct (> 35) or negative	Positive with low Ct (< 25–35)	Moderate ‒ positive in ∼74% when BI is negative^92^
Serology (anti-PGL-1)	Low/declining titers	Persistently high or rising titers	Low ‒ quantitative assays rarely available
**Note:** Always document the BI at treatment completion for future comparison. PCR and anti-PGL-1 alone do not confirm relapse, but strengthen suspicion when the clinical picture is compatible.			

Paucibacillary (PB)			
Slit-skin smear (BI)	Always negative	Always negative	None ‒ does not differentiate
**Histopathology**	Interstitial edema; intensified granulomatous response; perineural fibrinoid necrosis	Stable granuloma without acute changes	Moderate ‒ reactional changes are specific
**PCR**	Negative or Ct > 35	Ct < 30 (high load)	Moderate ‒ guides decisions when positive
**Serology (anti-PGL-1)**	Usually negative	Usually negative	None ‒ sensitivity < 30% in PB^54^
**Note:** Diagnosis relies on time since treatment completion + clinical course + response to empirical corticosteroids (up to 30-days for cutaneous lesions; up to 90 days for neuritis).			

BI, Bacteriological Index; Ct, Cycle threshold; MB, Multibacillary; PB, Paucibacillary.

## Conclusion

Complementary diagnostic tests are valuable in leprosy diagnosis, each with defined indications and limitations. Slit-skin smear microscopy and histopathology are most informative in advanced multibacillary disease; in early presentations ‒ when prompt diagnosis is critical ‒ their sensitivity is limited. Molecular methods may enhance detection in selected cases, although availability remains limited.

For neural assessment, Semmes-Weinstein monofilament testing remains the preferred method because of its practicality and reproducibility, and should be used routinely. The histamine test complements autonomic evaluation and is particularly useful in indeterminate forms. Nerve conduction studies/electromyography and ultrasonography provide additional information, where available, but do not replace serial clinical assessment. Recent magnetic resonance imaging studies have described spinal cord involvement, together with alterations in the dorsal root ganglia and the brachial plexus, broadening the recognized spectrum of neural involvement, although the diagnostic role of MRI remains under investigation.^52^, ^96^

No single laboratory test confirms the diagnosis of early leprosy. Clinical dermato-neurological judgment ‒ supported by simple functional tests such as monofilament testing and the histamine test ‒ remains decisive, with all other tests interpreted within the clinical-epidemiological context.

## ORCID IDs

Mirian Nacagami Sotto: 0000-0001-6380-7192

José Antônio Garbino: 0000-0002-4042-5797

João Avancini: 0000-0003-3038-6373

Maria Ângela Bianconcini Trindade: 0000-0003-1011-766X

## Financial Support

None declared.

## Research data availability

The entire dataset supporting the results of this study was published in this article.

## Authors' contributions

**Luis Alberto Ribeiro Fróes:** Conceptualization, Data curation, Methodology, Formal analysis, Investigation, Validation, Visualization, Writing - original draft. **Mirian Nacagami Sotto:** Data curation, Writing - review & editing. **José Antônio Garbino:** Data curation, Writing - review & editing. **João Avancini:** Data curation, Writing - review & editing. **Maria Ângela Bianconcini Trindade:** Supervision, Writing - review & editing.

## Conflicts of interest

None declared.
